# Probiotic and antibacterial properties of recombinant *Lactococcus lactis* expressing the fusion antimicrobial peptides BMAP18-BSN37 in mice and chickens

**DOI:** 10.1016/j.psj.2026.106507

**Published:** 2026-01-23

**Authors:** Ruibiao Wang, Yukai Lin, Yu Xia, Suxian Liu, Doudou Feng, Siyang Li, Tengyue Zhou, Huarun Sun, Jiyuan Shen, Bo Wen, Minghui Li, Chengshui Liao, Baoliang Qin, Jianhe Hu, Yuanfang Ma, Ke Ding, Lei Wang

**Affiliations:** a College of Animal Science and Veterinary Medicine, Henan Institute of Science and Technology, Xinxiang 453003, China.; b School of Basic Medical Sciences, Henan University, Zhengzhou, China.; c Ministry of Education Key Laboratory for Animal Pathogens and Biosafety, Zhengzhou 450000, China.; d Laboratory of Functional Microbiology and Animal Health, Henan University of Science and Technology, Luoyang 471023, China.; e Xinxiang Animal Disease Prevention and Control Center, Xinxiang 453003, China**.**

**Keywords:** Antimicrobial peptide BMAP18-BSN37, Recombinant *L. lactis* NZ-BB, Probiotics, Foodborne pathogens, Gut barrier integrity

## Abstract

Antimicrobial resistance poses a serious threat to global food safety and poultry production, prompting the need for effective alternatives to conventional antibiotics in food-producing animals. In this study, a recombinant food-grade strain, *L. lactis* NZ-BB, was engineered to express a fusion antimicrobial peptide (BMAP18-BSN37), and evaluated its probiotic characteristics and antimicrobial efficacy against *Salmonella*, a major foodborne pathogen in chicken. The recombinant plasmid pUBB was successfully constructed and introduced into *L. lactis* NZ9000, with optimal peptide expression achieved following Nisin induction (20 ng/mL, 6 h). NZ-BB demonstrated stable plasmid maintenance, high expression levels, and no detectable metabolic burden. *In vivo* trials using BALB/c murine and 817 strain avian models showed that NZ-BB enhanced body weight gain, supported immune organ development, and improved intestinal barrier integrity through upregulation of tight junction proteins (occludin, claudin-1, ZO-1) and anti-inflammatory cytokines (TGF-β, IL-4), while reducing pro-inflammatory markers (IL-1β, TNF-α, IL-17a). Importantly, oral administration of NZ-BB significantly reduced intestinal and systemic *Salmonella* burdens, mitigated tissue damage, and restored immune balance in both mice and chicks. Furthermore, NZ-BB regulated the expression of innate immune receptors (e.g., NLRC3) and matrix metalloproteinases (e.g., MMP-1), highlighting its immunomodulatory potential. These results underscore the dual probiotic and antimicrobial functionality of NZ-BB and support its potential use as a food-safe microbial agent to improve animal health and reduce the risk of *Salmonella* contamination in the food chain.

## Introduction

The overuse and misuse of antibiotics have fueled the emergence of “superbugs” by promting the horizontal and vertical transmission of drug-resistant genes, resulting in an escalating crisis in which conventional antibiotics are increasingly ineffective (Higuera-Llanten et al., 2018, Wang et al., 2024). This rapid escalation in bacterial resistance has galvanized the urgent need for alternative therapeutic strategies, prompting active research into new antimicrobial agents and novel treatments to counteract the growing resistance threat.

Antimicrobial peptides (AMPs) represent a diverse class of small peptides with potent antimicrobial properties. Upon exposure to pathogenic microorganisms, the host’s immune system rapidly synthesizes AMPs as part of its innate defense mechanisms, providing an immediate response to microbial invasion (Li et al., 2022). AMPs have demonstrated broad-spectrum antimicrobial activity, effective against a variety of pathogens, including bacteria, fungi, parasites, viruses, and even tumor cells. Additionally, they exhibited significant anti-inflammatory and immunomodulatory effects, making them highly attractive as potential alternatives to conventional antibiotics (Gani et al., 2025, Chatterjee and Sivashanmugam, 2024). However, natural AMPs often faced limitations, including relatively low antimicrobial potency, high cytotoxicity, and susceptibility to rapid degradation within the host, while synthetic AMPs were hindered by challenges such as high production costs, platform dependency, and long development timelines (Dong et al., 2018). To overcome these barriers, the development of efficient biological expression systems, facilitated by genetic engineering techniques, has emerged as a key strategy for the large-scale production of AMPs with enhanced stability, efficacy, and cost-effectiveness (Lei et al., 2021).

*L. lactis*, a food-grade intestinal probiotic, is non-cytotoxic and plays a crucial role in stimulating mucosal immunity, thereby enhancing the overall immune function of the host (Wu et al., 2023). Due to its safety profile and immunomodulatory properties, *Lactococcus* have gained significant attention as “protein factories” for the production of bioactive compounds. These microorganisms could be engineered to express and secrete heterologous AMPs, which could be consumed alongside the probiotics, providing a direct and effective delivery system (Tian et al., 2023, Feito et al., 2023). This approach offers a promising solution to the challenges associated with traditional AMP production methods, such as low yield and high costs. For instance, Volzing et al. successfully transferred A3APO and Alyteserin into *Lactobacillus casei* IL1403. The engineered strains produced and secreted these peptides, which demonstrated up to a 20-fold increase in antimicrobial activity against pathogenic *Escherichia coli* and *Salmonella* (Volzing et al., 2013). Similarly, Tanhaeian et al. expressed the antimicrobial peptide cLFchimera using the *Lactobacillus* P170, highlighting its strong anticancer properties (Tanhaeian et al., 2019). These studies underscore the effectiveness of *L. lactis*-based expression systems in producing active AMPs, which not only offer antimicrobial benefits but also have potential therapeutic applications.

Bovine cathelicidin-derived antimicrobial peptide BMAP-18 exhibits broad-spectrum antimicrobial activity, effectively targeting a wide range of pathogens, including bacteria, fungi, and viruses (Yang et al., 2019, Jahan et al., 2023). Additionally, the antimicrobial peptide BSN-37, derived from bovine neutrophils, represents a truncated and naturally active form of the antimicrobial peptide Bac5. BSN-37 has demonstrated potent antibacterial effects against gram-negative enteric bacteria, exhibiting minimal hemolysis and no detectable cytotoxicity (Yang et al., 2020a).

*L. lactis* is a food-grade bacterium with a long history of safe use, and the species is generally recognized as safe (GRAS) (Linares et al., 2010). Owing to its well-characterized genetics and established application in biotechnology, *L. lactis* NZ9000 was selected as the host strain in this study. The use of *L. lactis* as a feed-related microbial strain is consistent with the safety principles applied by regulatory frameworks in the USA, the EU, and China, including the FDA/AAFCO feed safety framework, EFSA FEEDAP guidelines under Regulation (EC) No. 1831/2003 (Anadón et al., 2006), and China’s GB 7300.502-2023 standard for feed additives. These frameworks emphasize clear strain identification, absence of pathogenicity, and comprehensive safety evaluation, providing a regulatory basis for the further assessment of recombinant strains intended for zootechnical applications. The aim of this study is to construct a recombinant *L. lactis* NZ9000 strain capable of co-expressing BMAP-18 and BSN-37, evaluate the antibacterial potential and application prospects and ultimately develop a novel probiotic preparation. This preparation is intended to serve as an antibiotic alternative, thereby providing a new strategy for the sustainable and healthy development of the animal husbandry industry.

## Materials and methods

### Animals, strains, and plasmids

Experimental Animals: Eight-week female BALB/c mice were purchased from HFK Bio-Technology.co.,LTD (China), and One-day-old broiler chicks (817 strain) were obtained from Henan Province, China. All experimental animals were free from *Salmonella* infection. All animal experiments and related procedures were conducted in strict accordance with the “Regulations for the administration of laboratory animals” and the guidelines of the Animal Management Committee of Henan Institute of Science and Technology (Ethics Approval No. LLSC2024052). Throughout the study, both mice and chicks had free access to food and water and were maintained in SPF (Specific Pathogen Free) conditions. The environmental humidity was maintained at 70 % ± 10 %, and the temperature was kept at 20 °C ± 2 °C to ensure the health of the animals and the accuracy of the experiments.

Strains: *L. lactis* NZ9000 strain was purchased from Miaoling Bio (China). *Salmonella enterica serovar Typhimurium* strain CVCC541 and *Salmonella enterica serovar Pullorum* (SP) were preserved in the research team of “animal pathogen and new veterinary drug” at Henan institute of science and technology. Plasmids: Vector pNZ8148 was cloned into competent MC1061 cells and stored at -80 °C.

### Construction and identification of recombinant plasmid pNZ-BB

The amino acid sequences of the antimicrobial peptides BMAP-18 and BSN-37 were obtained from the APD3 database (APD_ID: AP03843 for BMAP-18 and AP03803 for BSN-37). Codon optimization for the CDs was performed using an online tool for codon optimization (https://www.genscript.com.cn/), tailored to the codon usage preference of *L. lactis*. Gene design: The BMAP-18 and BSN-37 sequences were tandemly repeated twice, with a linker (GGGGS)_3_ inserted between them to form the target sequence for the fusion antimicrobial peptide BMAP18-BSN37. A 5’ homologous arm of pNZ8148, an Nco I restriction site, and the Usp45 signal peptide were added upstream of the fusion peptide, while a His tag, Hind III restriction site, and a 3’ homologous arm of pNZ8148 were added downstream. The target gene (611 bp in length) was synthesized by Zoonbio Bio (China) and cloned into the plasmid pUC57. The plasmid pUC57-BMAP18-BSN37 served as the template for amplification of the Usp45-BMAP18-BSN37 fragment using Pfu high-fidelity enzyme (Beyotime, China). Primer sequences were listed in Table 1. Plasmid pNZ8148 was double-digested with Nco I and Hind III (Takara, Japan), and the linearized pNZ8148 plasmid was recovered by gel extraction. Homologous recombination of the Usp45-BMAP18-BSN37 gene with the linearized pNZ8148 plasmid was carried out using the ClonExpress II One Step Cloning Kit (Vazyme, China), resulting in the recombinant plasmid pNZ8148-Usp45-BMAP18-BSN37 (abbreviated as pUBB).

**Table 1:** Primers used in the construction of *L. lactic* NZ-BB.

**Name**	**Primers**	**Sequence**	**Sequence length**
Usp45-BMAP18-BSN37	Forward	5’- taaggaggcactcaccatgggcatgaaaaaaaagattatc -3’	610 bp
	Reverse	5’- attttggttcaaagaaagcttttagtgatgatgatgatg -3’	
pNZ-BB	Forward	5’- GTGATGGTTATCATGCTGGATTG -3’	842 bp
	Reverse	5’- TAGGTGGACGACGGATAGGA -3’	

### Transformation of plasmid into *L. lactis* NZ9000

Plasmids pUBB and pNZ8148 were mixed with competent cells of NZ9000 and transferred into a pre-chilled 0.2 cm electroporation cuvette (Bio-Rad, USA), respectively. The mixture was incubated for 5 min. Electroporation was performed using a Gene Pulser Xcell electroporator (Bio-Rad, USA) with the following parameters: 2000 V, 25 μF, 200 Ω, with a single pulse. After electroporation, 1 mL GM17-MC medium was immediately added to the mixture for recovery for 2 h. The cells were then plated onto GM17 agar plates containing 10 μg/mL chloramphenicol (Chl). Positive transformants, identified as recombinant strains, were NZ9000-pNZ8148 (NZ-pNZ), containing the empty plasmid pNZ8148, and NZ9000-BMAP18-BSN37 (NZ-BB), containing the plasmid pUBB.

### Isolation of recombinant strain NZ-BB

PCR identification of positive transformants was performed, yielding an amplicon of 842 bp (primer sequences were listed in Table 1). PCR-positive transformants were cultured to an OD_600_ of 0.4, followed by the addition of Nisin (20 ng/mL) and continued incubation for 6 h to induce target gene expression. After induction, the culture was centrifuged at 12,000 rpm for 2 min to separate the bacterial pellet from the supernatant. The bacterial pellet was then processed by sonication, and the resulting cell lysate was collected. Western blot analysis was performed to detect the expression of the target protein BMAP18-BSN37, using an anti-His antibody (ZSGB-Bio, China) as the primary antibody, with the target protein expected to be approximately 20 kDa. The strain with the highest expression of BMAP18-BSN37 was selected as the target for subsequent experimental studies. Different induction times (2, 4, 6, 8, 10, and 12 h) and Nisin concentrations (0, 5, 10, 15, 20, 25, 30, and 35 ng/mL) were tested to optimize the best induction conditions and concentration using Western blot analysis.

### Evaluation of the biological properties of recombinant strains

Growth curve: The strains were divided into four groups: NZ-pNZ, NZ-BB, NZ-pNZ + Nisin, and NZ-BB + Nisin, with an initial OD_600_ of 0.01. For the two Nisin-induced groups, Nisin was added when the OD_600_ reached approximately 0.4. The OD_600_ values of the different bacterial cultures were measured every 0.5 h, with the experiment repeated three times to generate the growth curves.

Plasmid stability: The recombinant strain NZ-BB, cultured to the stationary phase in medium without Chl, was serially subcultured at a 1:10 dilution ratio every 8 h, with each passage representing one generation. The culture was subcultured for up to 80 generations, and every 5 generations, the bacterial cultures were subjected to PCR to verify the presence of the plasmid pUBB.

### Animal grouping and treatment

#### Probiotic treatment grouping and administration

BALB/c mice were randomly assigned to three experimental groups: 0.9 % NaCl, NZ-pNZ (vehicle control), and NZ-BB. The two *L. lactis* strains, NZ-pNZ and NZ-BB, were administered orally at a dose of 1 × 10^9^ colony-forming units (CFU) per mouse in 1 mL suspension, twice daily for 30 consecutive days. One-day-old healthy “817” broiler chicks with uniform body weight were randomly allocated into the same experimental groups and subjected to identical treatment protocols as the mice, with the intervention lasting for 14 consecutive days.

#### Antimicrobial grouping and treatment

**Fig. 1:**
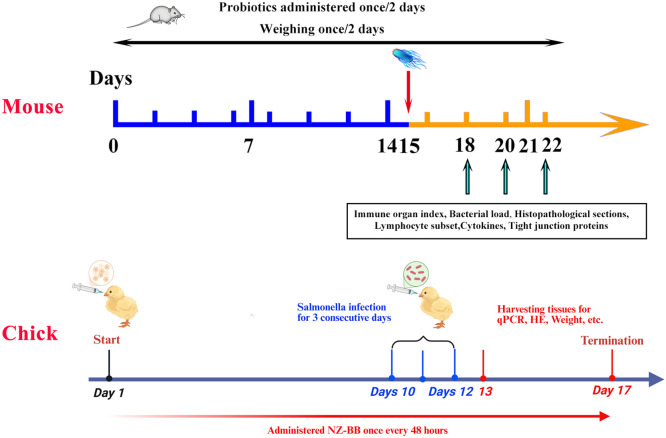
Schematic diagram of the animal experiments.

BALB/c mice were randomly assigned to four experimental groups: 0.9 % NaCl, 0.9 % NaCl + CVCC541 (the positive control), NZ-pNZ + CVCC541, and NZ-BB + CVCC541. The administration of *L. lactis* strains and 0.9 % NaCl was conducted via oral gavage, with each *L. lactis* strain provided at a concentration of 1 × 10^9^ CFU, twice daily, until the conclusion of the study, at a dosage of 1 mL. The *Salmonella Typhimurium* CVCC541 strain was delivered intraperitoneally at a concentration of 1 × 10^7^ CFU, with a volume of 250 μL. On the 15th day, the mice were challenged with CVCC541, and pertinent parameters were assessed on days 3, 5, and 7 following infection. The experiment began with 3-day-old “817” chicks, using the same *L. lactis* grouping and administration as the mouse study. On day 10, chicks were infected with SP for three days at 2.5 × 10^8^ CFU and 250 μL. Parameters were evaluated on the 3rd and 7th days after infection. The schematic diagram of animal experiments is shown in Fig. 1.

### Assessment of growth performance and indices of immune organs

Growth Performance: The body weight of each cohort of mice and chicks was systematically recorded at two-day intervals. Prior to each weighing session, the animals underwent an 8 h fasting period, during which they had unrestricted access to water. Indices of Immune Organs: At predetermined time points, the thymus, spleen, and mesenteric lymph nodes were excised from the mice, whereas the spleen, mesenteric lymph nodes, and bursa of Fabricius were collected from the chicks . The immune organ index was subsequently calculated for each group.

### Flow cytometry

Spleens from each group were immersed in PBS, and single-cell suspensions were prepared by grinding the tissue through a 200-mesh sieve. Erythrocytes were lysed using red blood cell lysis buffer (Biyotime, China), and lymphocytes were collected and resuspended in flow cytometry buffer at 1 × 10^7^ cells/mL. Subsequently, 100 μL of the cell suspension was stained with antibodies for surface marker detection, specifically FITC Hamster Anti-mouse CD3e, APC Rat Anti-Mouse CD4, and PE Rat Anti-Mouse CD8a (BD Pharmingen, USA). Negative controls (no antibody), single-staining controls for each of the three antibodies, and experimental samples were prepared. For each sample, 1 μg of each antibody was added and incubated in the dark for 15 min. Following incubation, 1 mL of flow cytometry buffer was added to each tube, and the samples were centrifuged at 4 °C at 300 g for 5 min. The supernatant was discarded, and the cells were resuspended in 500 μL of flow cytometry buffer for subsequent analysis by flow cytometry.

### RT-qPCR

Total RNA was isolated from the jejunal tissues of both mice and chicks utilizing the RNAeasy™ Animal RNA Extraction Kit (Biyotime, China). The concentration and purity of the RNA samples were evaluated using the NanoDrop 2000 spectrophotometer (Thermo Fisher, USA). Subsequently, the isolated total RNA was reverse transcribed into cDNA employing the HiScript II 1st Strand cDNA Synthesis Kit (Vazyme, China). RT-qPCR was conducted to assess the relative expression levels of target genes using the ChamQ Universal SYBR qPCR Master Mix (Vazyme, China) in conjunction with the Roche LightCycler 480 system. GAPDH served as the reference gene, and the relative expression levels of the genes of interest were determined using the 2^−∆∆Ct^ method. The primer sequences utilized for mice and chicken samples were detailed in Table 2, Table 3.

**Table 2:** Primers of the detected genes in jejunal tissue of mice.

**Gene name**	**Primers**	**Sequence**	**Access Number**
GAPDH	Forward	5’- GTGAAGGTCGGTGTGAACGGATT-3’	NM_001289726.2
	Reverse	5’- GGTCTCGCTCCTGGAAGATGGT-3’	
ZO-1	Forward	5’- TCGTCTGTCCTACCTGTC-3’	NM_009386.3
	Reverse	5’- CTGTATCTGTGTCTTCATAGTC-3’	
occludin	Forward	5’-GGCTGCTGCTGATGAATA-3’	NM_008756.2
	Reverse	5’-ATCCTCTTGATGTGCGATAA-3’	
claudin-1	Forward	5’-AGATGTGGATGGCTGTCA-3’	NM_016674.4
	Reverse	5’-TCATACCTGGCATTGATGG-3’	
E-Cadherin	Forward	5’-GACTTAGAGATTGGCGAATAC-3’	NM_009864.3
	Reverse	5’-GAGGATGGCAGGAACTTG-3’	
IL-1β	Forward	5’-CTTCAGGCAGGCAGTATC-3’	NM_008361.4
	Reverse	5’-CAGCAGGTTATCATCATCATC-3’	
IL-4	Forward	5’- GCCATATCCACGGATGCGACAA-3’	NM_021283.2
	Reverse	5’- GGTGTTCTTCGTTGCTGTGAGG-3’	
IL-6	Forward	5’- TCCATCCAGTTGCCTTCT -3’	NM_031168.2
	Reverse	5’-TAAGCCTCCGACTTGTGA-3’	
IL-17α	Forward	5’- GATGCTGTTGCTGCTGCTGAG-3’	NM_010552.3
	Reverse	5’- CGTGGAACGGTTGAGGTAGTCT-3’	
TGF-β	Forward	5’-GCAACAACGCCATCTATG-3’	NM_011577.2
	Reverse	5’-CAAGGTAACGCCAGGAAT-3’	
TNF-α	Forward	5’-GTGGAACTGGCAGAAGAG-3’	NM_001278601.1
	Reverse	5’-GAGAAGAGGCTGAGACATAG-3’

**Table 3:** Primers of the detected genes in jejunal tissue of chicks.

**Gene name**	**Primers**	**Sequence**	**Access Number**
ZO-2	Forward	5’- GTGATGGAGAGGAGGAGGAGGAGTA-3’	NM_001396726.1
	Reverse	5’- CACAGACCAGCAAGCCTACAGTTC-3’	
claudin-1	Forward	5’- GGAGGATGACCAGGTGAAGAAGATG-3’	NM_001013611.2
	Reverse	5’- CCGAGCCACTCTGTTGCCATAC-3’	
E-Cadherin	Forward	5’- AAGGCACAGGTGACGCAGGT-3’	NM_001039258.3
	Reverse	5’- AGCAGCAGCAGCAGCAGGAT-3’	
IL-1β	Forward	5’- GGCACAGAGATGGCGTTCGT-3’	NM_204524.2
	Reverse	5’- GAATCCAGGCGAGGCTTCTTCT-3’	
IL-2	Forward	5’- GGCTAACTAACCTGCTGTCCATTCT-3’	NM_204153.2
	Reverse	5’- CCGTAGGGCTTACAGAAAGGATCAA-3’	
IL-4	Forward	5’- AGGAGAGGCGAGCGATGGAA-3’	NM_001007079.2
	Reverse	5’- TAACAGTGGTAGGAGGCAGATGGT-3’	
IL-6	Forward	5’- AGGCTGAAGAACTCCACTGTATCCA-3’	NM_204628.2
	Reverse	5’- GTTCAACCTCTGCTGCCATTCCA-3’	
IL-17A	Forward	5’- GCCATTCCAGGTGCGTGAACT-3’	NM_204460.2
	Reverse	5’- TCTTCTCCAGGCGGTACGAGTG-3’	
TGF-β1	Forward	5’- GCCGACACGCAGTACACCAA-3’	NM_001318456.1
	Reverse	5’- GCAGGCACGGACCACCATATTG-3’	
TNF-α	Forward	5’- ACCACGAGTAGGATGTCTGTAGAGG-3’	NM_204267.2
	Reverse	5’- CGAGCAACTGCCAGCCACTT-3’	
MUC-1	Forward	5’- GGTGTCCAGAAGCAGCAGATGTG-3’	XM_015279046.4
	Reverse	5’- GCAGCAGATGTGAGCAGTGATGT-3’	
iNOS	Forward	5’- GTGGTATGCTCTGCCTGCTGTT-3’	NM_204961.2
	Reverse	5’- AAGTCTCGCACTCCAATCTCTGTTC-3’	
MMP1	Forward	5’- AGGTCAGGACTTCGCAGTGTAGC-3’	XM_417176.7
	Reverse	5’- GCAGCATACAACAGCAGGAGAAGAG-3’	
MMP2	Forward	5’- AACAGAAGGCAGGACAGATGGATAC-3’	NM_204420.3
	Reverse	5’- CCACTTGCGGTCATCATCATAGC-3’	
MMP9	Forward	5’- GTGCCGTGATAGATGATGCCTTCC-3’	NM_204667.2
	Reverse	5’- TGTCTGCCTCGCCGCTGTAA-3’	
NOD1	Forward	5’- GCAATCAGGTTGGAGACGAAGGT-3’	NM_001318438.1
	Reverse	5’- CGTGATGCCATTGAATGCGAGAC-3’	
NLRC3	Forward	5’- GGAGGAAGCGATGAAGAACGAGAG-3’	XM_015294675.4
	Reverse	5’- GTTGTAAGTGAGGCAGTTGGAGAGG-3’	
GAPDH	Forward	5’- GGCACGCCATCACTATCTTCCA-3’	NM_204305.2
	Reverse	5’- GACTCCACAACATACTCAGCACCT-3’	

### Western blot

Total protein was extracted from the jejunal tissue of mice utilizing RIPA Lysis Buffer (Strong) (Yeason, China). Protein concentrations were quantified via the BCA assay. Proteins were then resolved by 12.5 % SDS-PAGE using the Color PAGE Gel Rapid Preparation Kit (Yamei, China) and subsequently transferred onto a 0.45 μm PVDF membrane (Millipore, USA). The membrane was blocked with 5 % skim milk at ambient temperature for 2 h and then incubated overnight at 4 °C with a primary antibody (Boster, China). Following washes with PBST, the membrane was incubated with an HRP-conjugated secondary antibody for 2 h. Detection of protein bands was achieved using an ECL reagent (Meilunbio, China), and the bands were visualized. Quantification of the bands was performed using a chemiluminescence imaging system (Syngene, USA) and Image J software.

### Bacterial load and H&E staining of tissues

At 3, 5, and 7 days post-infection (DPI) with CVCC541, the liver, spleen, and jejunum of mice were aseptically excised. Similarly, at 3 and 7 days post-infection with SP, the liver, spleen, and jejunum of chicks were aseptically collected. Tissue samples weighing 1 gram from each group were homogenized using a tissue homogenizer operating at 60 Hz for 2 min. The homogenized tissue samples were subsequently diluted to a 10^-3^ concentration, and 100 μL of the resulting homogenate were plated onto Salmonella Shigella (SS) agar plates. These plates were incubated at 37 °C for 16 h, after which the number of *Salmonella* colonies present in the various tissues was enumerated.

Tissue samples including the jejunum, liver, and spleen from mice, along with the jejunum from chicks, were collected for histopathological examination. Following fixation, tissues were processed for hematoxylin and eosin (H&E) staining. Histopathological sections were prepared and examined under light microscopy to evaluate tissue morphology and pathological lesions.

### Statistical data analysis

All statistical results were presented as the mean ± SEM from three independent experiments. Statistical significance was assessed using one-way ANOVA in SPSS software. Graphical representations were created using GraphPad Prism software. Western blot results were quantified using ImageJ software. A p-value of < 0.05 was considered statistically significant.

## Results

### Screening of recombinant *L. lactis* expressing the antimicrobial peptide BMAP18-BSN37

**Fig. 2:**
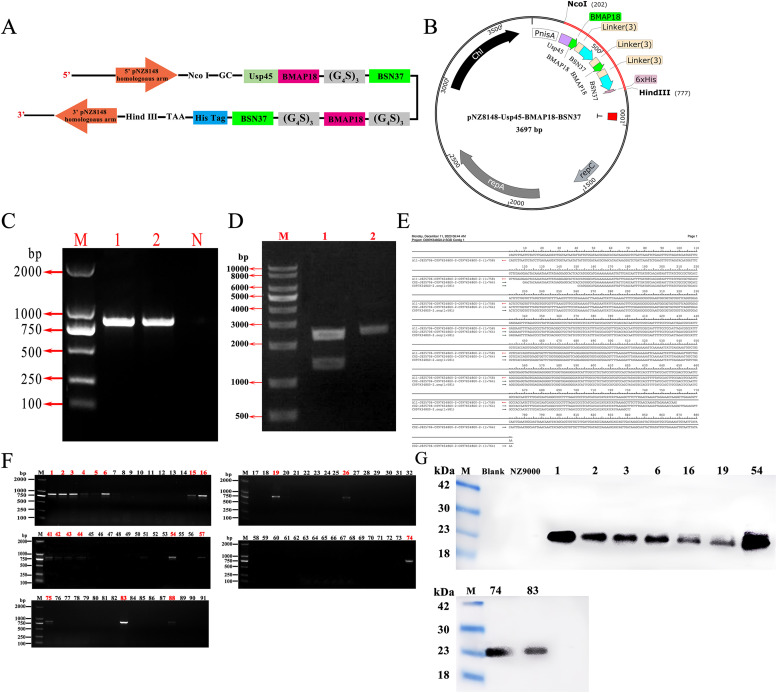
**Screening of recombinant *L. lactis* strains expressing the fusion antimicrobial peptide BMAP18-BSN37.** (A) Schematic diagram of the fusion protein BMAP18-BSN37 design. (B) Diagram of the construction of the recombinant expression vector pUBB. (C) PCR identification of the fusion antimicrobial peptide BMAP18-BSN37 (842 bp). M: DNA ladder (2000 bp); lanes 1 and 2: target gene; N: negative control. (D) Double digestion analysis of plasmid pUBB using Hind III and Nde I. M: DNA ladder (10,000 bp); lane 1: plasmid digested with HindIII and NdeI; lane 2: undigested plasmid. (E) Sequencing alignment of BMAP18-BSN37. (F) PCR screening of positive colonies transformed with pUBB. Lanes 1-91: Numbers of positive transformants identified. (G) Western blot screening of recombinant strains expressing BMAP18-BSN37. Lanes 1, 2, …, 74, 83: recombinant strains expressing BMAP18-BSN37.

The antimicrobial peptide BMAP18-BSN37 was designed, with a schematic representation shown in Fig. 2A. Through homologous recombination, the BMAP18-BSN37 fragment was inserted into the linearized pNZ8148 vector to construct the recombinant expression plasmid pUBB (Fig. 2B). PCR amplification confirmed the presence of an 842 bp gene fragment, matching the expected size (Fig. 2C). Restriction enzyme digestion with Hind III and Nde I yielded target fragments of 2535 bp and 1162 bp, respectively (Fig. 2D). Sequence alignment via BLAST analysis demonstrated 100 % homology between the sequencing result and the intended fragment (Fig. 2E). The recombinant plasmid pUBB was subsequently electroporated into *L. lactis* strain NZ9000, generating the recombinant strain NZ-BB. PCR screening of 91 positive transformants identified 20 colonies successfully amplifying the target fragment (842 bp) (Fig. 2F). Following Nisin induction, the Western blot analysis of cell lysates from these 20 colonies revealed a protein band at the expected molecular weight of 20 kDa in 7 strains (Fig. 2G). Among these, the strain exhibiting the highest expression of the exogenous BMAP18-BSN37 peptide was selected for further studies and designated as NZ-BB.

### Performance evaluation of recombinant *L. lactis* NZ-BB

**Fig. 3:**
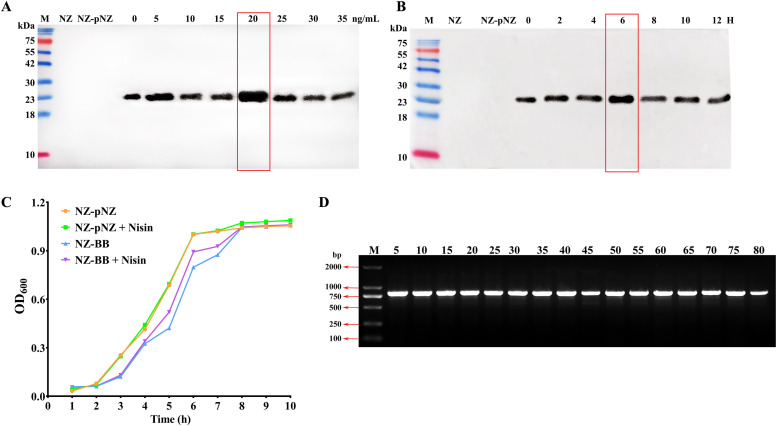
**Screening of recombinant *L. lactis* strains expressing the fusion antimicrobial peptide BMAP18-BSN37.** Optimization of (A) inducer concentration and (B) induction time. (C) Growth curve measurement of recombinant strains. (D) Stability of plasmid pUBB in NZ-BB. Lanes 5, 10, …, 80: Generations.

To optimize the expression of the exogenous protein BMAP18-BSN37, different Nisin concentrations and induction times were evaluated. As shown in Fig. 3A and 3B, the maximal protein expression was achieved at 20 ng/mL Nisin with 6 h of induction. To assess whether recombinant protein expression affected bacterial growth, the growth kinetics of the recombinant strain NZ-BB were compared with those of the control strain NZ-pNZ (harboring vector pNZ8148). Growth curves (Fig. 3C) showed that both strains exhibited similar trends under static culture at 30 °C, with a lag phase (0-2 h), a logarithmic growth phase (2-6 h), and a plateau phase (beyond 6 h). Notably, Nisin induction did not alter the growth dynamics, indicating that BMAP18-BSN37 expression did not impose a metabolic burden on the host strain. To evaluate plasmid stability, NZ-BB was continuously passaged in antibiotic-free GM17 medium for 80 generations (with subculturing every 8 h). PCR-based monitoring of the pNZ-UBB plasmid at 5-generation intervals confirmed its stable retention in *L. lactis* throughout the 80 generations (Fig. 3D).

### Probiotic potential and safety evaluation of recombinant strain NZ-BB in mice

**Fig. 4:**
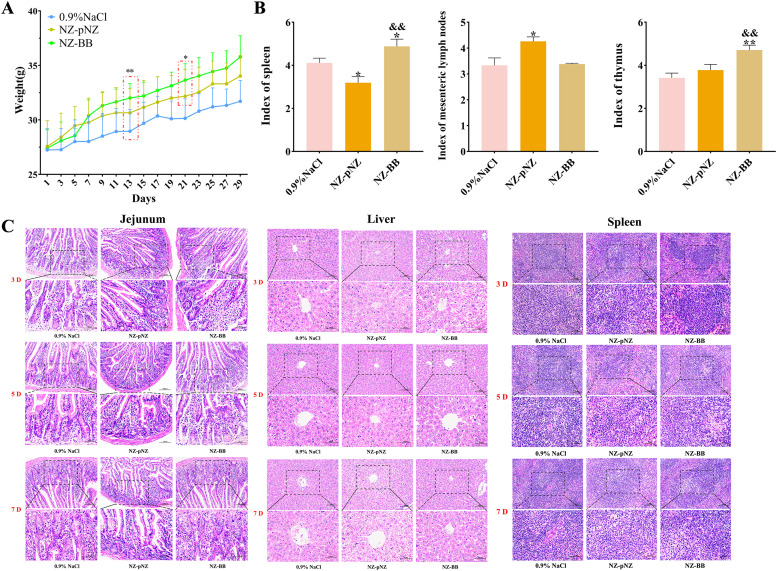
**Probiotic potential and safety evaluation of NZ-BB in mice.** (A) Variation of body weight. (B) Immune organ indices. From left to right: spleen, mesenteric lymph nodes, and thymus. *P < 0.05, ^⁎⁎^*P* < 0.01, ^⁎⁎⁎^*P* < 0.001 vs. 0.9 % NaCl group; ^&^*P* < 0.05, ^&&^*P* < 0.01, ^&&&^*P* < 0.001 vs. NZ-pNZ group. (C) Histological analysis by H&E staining. Scale bars: 100 μm for 200× magnification; 50 μm for 400× magnification.

To evaluate the probiotic potential of recombinant strain NZ-BB, a 30-day mice study was performed with three treatment groups. Repeated measures ANOVA showed significantly greater weight gain in both *L. lactis* groups (NZ-pNZ and NZ-BB) compared to the 0.9 % NaCl control by day 13 (*P* < 0.01). Moreover, NZ-BB promoted significantly more weight gain than NZ-pNZ by day 21 (*P* < 0.05) (Fig. 4A). On day 7, immune organ indices were measured to assess immunomodulation (Fig. 4B). The spleen index decreased significantly in the NZ-pNZ group (*P* < 0.05), while the NZ-BB group showed a significant increase (*P* < 0.05). The mesenteric lymph node index increased in NZ-pNZ-treated mice (*P* < 0.05), whereas NZ-BB-treated mice showed levels comparable to controls. Notably, the thymus index was significantly higher in the NZ-BB group than in both NZ-pNZ and control groups (*P* < 0.01), suggesting enhanced immune function. Histological examination of the jejunum, liver, and spleen (Fig. 4C) revealed preserved tissue structure in NZ-BB-treated mice at days 3, 5, and 7. Jejunal villi remained intact with orderly epithelial cells and minimal inflammation. Liver tissue showed normal central vein structure and hepatocyte morphology without notable degeneration. Splenic architecture was well-defined, with no signs of inflammatory infiltration. Similar tissue integrity was observed in the NZ-pNZ group. These results indicate that NZ-BB administration maintains normal histology and supports immune health.

### Study of the probiotic mechanisms of recombinant strain NZ-BB in mice

**Fig. 5:**
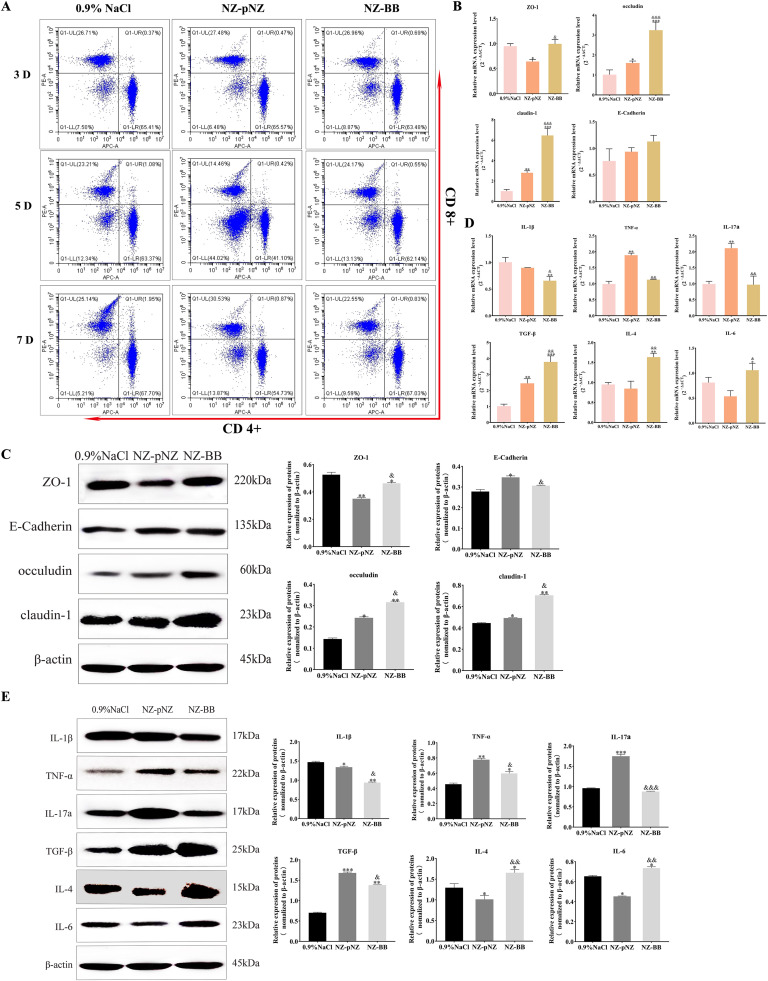
**Probiotic mechanisms of NZ-BB in mice.** (A) Flow cytometric profiling of T-cell subset distribution. Expression levels of tight junction proteins assessed by (B) RT-qPCR and (C) Western blot analysis. Inflammatory cytokine expression evaluated by (D) RT-qPCR and (E) Western blot. Note: Statistical significance is denoted as in Fig. 4.

Building upon the demonstrated probiotic potential and safety profile of NZ-BB (Section 3.2), mechanistic study revealed distinct immunomodulatory and barrier-enhancing properties. Flow cytometric analysis of splenic T cell populations showed no significant alterations in CD4+/CD8+ ratios across treatment groups or timepoints (3/5/7 days) (Fig. 5A and Table 4). Quantitative assessments of intestinal barrier function demonstrated NZ-BB-specific upregulation of both transcriptional (*P* < 0.001) and translational (*P* < 0.001) expression of occludin and claudin-1, exceeding levels observed in both NZ-pNZ and the control groups (Fig. 5, Fig. 5). While NZ-pNZ administration similarly enhanced these tight junction proteins versus 0.9 % NaCl, it paradoxically reduced ZO-1 mRNA expression. Cytokine profiling revealed NZ-BB’s unique immunoregulatory signature: suppressed IL-1β concomitant with elevated TGF-β, IL-4 and IL-6 (Fig. 5, Fig. 5), contrasting with NZ-pNZ-induced elevations in pro-inflammatory TNF-α and IL-17a.

**Table 4:** Flow cytometry analysis of T-cell subsets in probiotic-treated mice.

**Days**	**T cells (%)**	**0.9** % **NaCl**	**NZ-pNZ**	**NZ-BB**
**3**	CD^4+^	65.05	65.68	63.26
	CD^8+^	26.82	27.46	27.01
	CD^4+^/CD^8+^	2.426 ± 0.012	2.392 ± 0.009	2.342 ± 0.011
**5**	CD^4+^	63.99	41.28	63.21
	CD^8+^	23.11	14.40	25.01
	CD^4+^/CD^8+^	2.769 ± 0.025	2.867 ± 0.024	2.527 ± 0.038
**7**	CD^4+^	67.89	55.22	67.06
	CD^8+^	24.80	30.32	22.53
	CD^4+^/CD^8+^	2.737 ± 0.012	1.821 ± 0.014^a^	2.977 ± 0.003

Note: ^a^indicates a significant difference between the 0.9 % NaCl group and other groups (*P* < 0.05).

### Protective effect of recombinant strain NZ-BB against Salmonella infection in mice

**Fig. 6:**
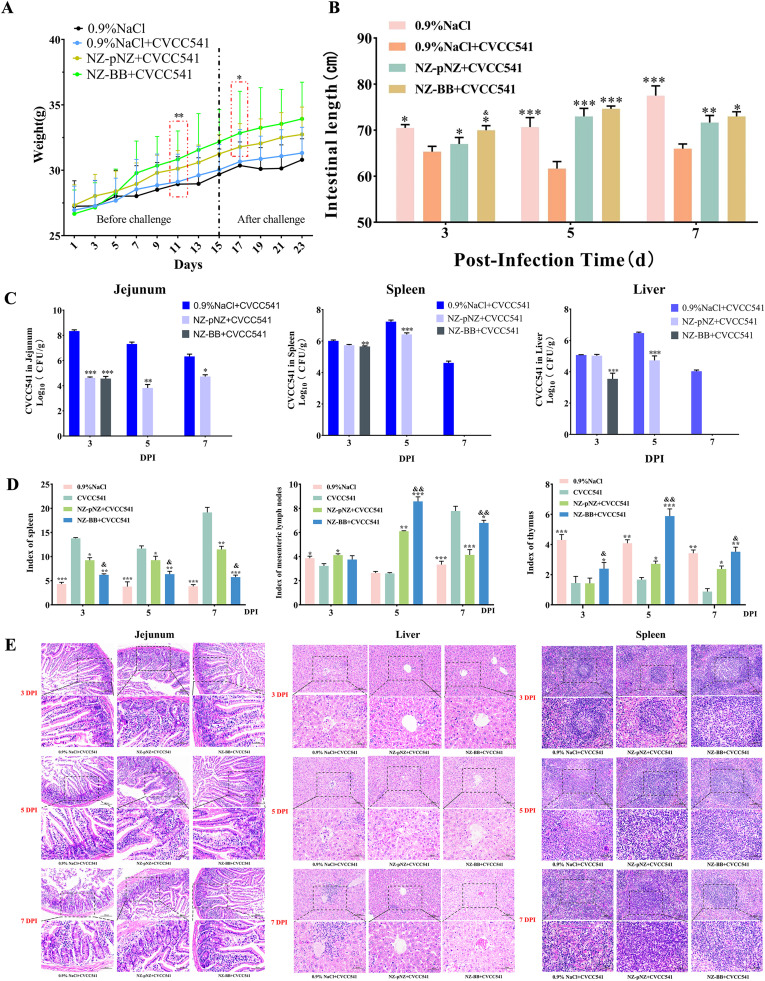
**Protective effect of NZ-BB on *Salmonella*-infected mice.** (A) Variation of body weight. (B) Length of small intestine. (C) Tissue bacterial load. (D) Immune organ indices. (E) Histological analysis by H&E staining. *P < 0.05, ^⁎⁎^*P* < 0.01, ^⁎⁎⁎^*P* < 0.001 vs. 0.9 % NaCl+CVCC541 group; ^&^*P* < 0.05, ^&&^*P* < 0.01, ^&&&^*P* < 0.001 vs. NZ-pNZ group.

In order to assess the protective efficacy of recombinant *L. lactis* NZ-BB against *Salmonella Typhimurium* CVCC541, a mouse model was established. Body weight monitoring revealed that both NZ-pNZ and NZ-BB significantly improved weight gain compared to controls from day 11 onwards (*P* < 0.01). Notably, mice in the NZ-BB + CVCC541 group showed significantly higher weight than those in the NZ-pNZ + CVCC541 group at day 17 (DPI 2; *P* < 0.05) (Fig. 6A). Protective effects were further assessed through measurements of jejunal length, bacterial load, immune organ indices, and tissue histopathology across DPI 3, 5, and 7. As shown in Fig. 6B, *Salmonella* infection markedly reduced jejunal length at all timepoints. Both NZ-pNZ and NZ-BB alleviated this shortening at DPI 3 and 5, with NZ-BB providing greater protection, particularly at DPI 3. Partial protection remained at DPI 7, though full recovery was not achieved. Bacterial quantification (Fig. 6C) indicated that both probiotics significantly reduced jejunal Salmonella colonization from DPI 3 onward (*P* < 0.001), with NZ-BB achieving full clearance by DPI 5. Similar reductions were seen in liver and spleen, where NZ-BB significantly lowered bacterial loads from DPI 3 (*P* < 0.01) and NZ-pNZ from DPI 5 (*P* < 0.001), with complete clearance by NZ-BB at DPI 5. Immune organ analysis (Fig. 6D) showed that both probiotics mitigated infection-induced splenomegaly across all timepoints, with NZ-BB being more effective at DPI 3 (*P* < 0.05). Mesenteric lymph node indices were not affected by infection alone at DPI 5, but both treatments increased lymph node size, with NZ-BB inducing a stronger response (*P* < 0.01). By DPI 7, infection-induced hypertrophy was reversed by both strains, with NZ-pNZ showing a slight advantage. Thymus atrophy caused by infection was significantly reversed by NZ-BB from DPI 3 (*P* < 0.05), while NZ-pNZ achieved similar effects from DPI 5. NZ-BB consistently performed better in restoring thymus weight. Histological analysis (Fig. 6E) revealed typical *Salmonella*-induced lesions at DPI 3, including jejunal villus damage, liver steatosis, and splenic structural disruption. Both NZ-pNZ and NZ-BB alleviated these lesions across all timepoints, with NZ-BB showing superior histological protection.

### Mechanisms of protection against Salmonella infection by recombinant strain NZ-BB

**Fig. 7:**
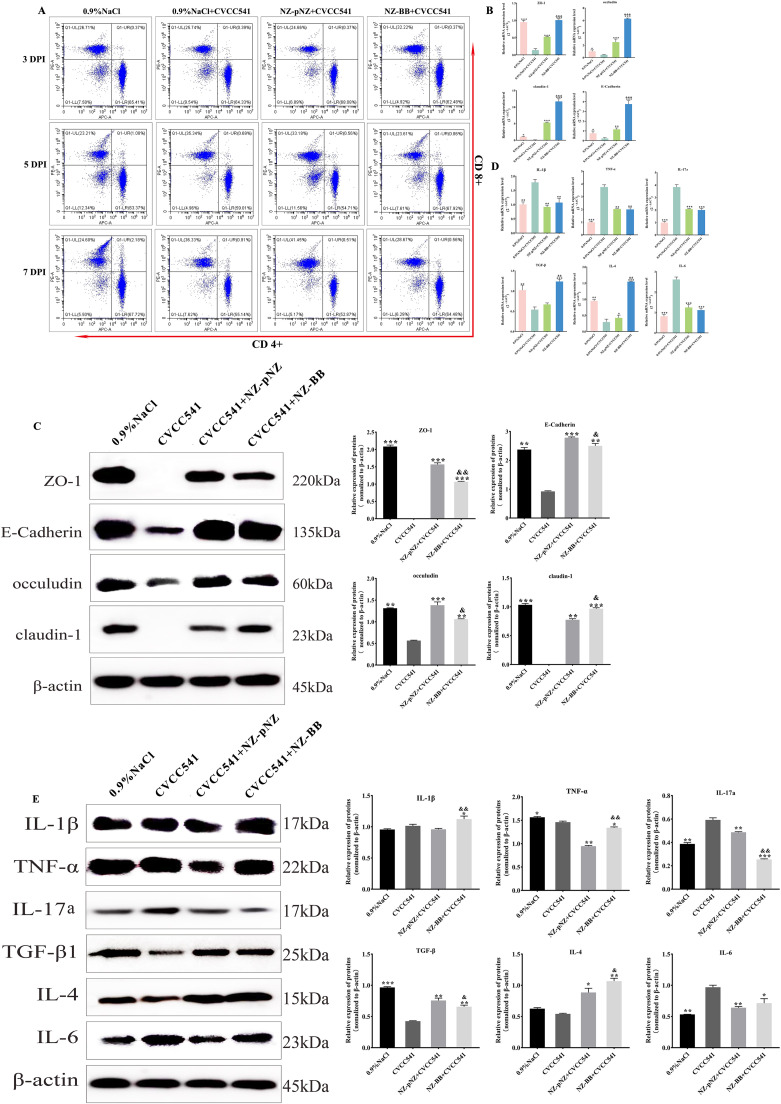
**Mechanisms of NZ-BB strain against Salmonella infection.** (A) Flow cytometric profiling of T-cell subset distribution in infected mice treated with NZ-BB. Expression levels of tight junction proteins assessed by (B) RT-qPCR and (C) Western blot analysis. Inflammatory cytokine expression evaluated by (D) RT-qPCR and (E) Western blot. Note: Statistical significance is denoted as in Fig. 6.

As demonstrated in Section 3.5, the recombinant strain NZ-BB exhibited protective efficacy against *Salmonella* infection in mice. To gain deeper insight into the protective mechanisms, further analyses were performed on T lymphocyte subsets, the expression of tight junction proteins and inflammatory cytokines in the jejunum. Flow cytometric analysis (Fig. 7A and Table 5) revealed no significant differences in the CD^4+^/CD^8+^ T cell ratios across all treatment groups at 3 DPI (*P* > 0.05). However, at DPI 5, both CVCC541 and NZ-pNZ groups exhibited significantly reduced CD^4+^/CD^8+^ ratios compared to the control and NZ-BB groups (*P* < 0.05), with this trend continuing through DPI 7. RT-qPCR and Western blot analyses at DPI 7 revealed that *Salmonella* infection significantly decreased both the transcriptional and translational levels of tight junction proteins (ZO-1, occludin, claudin-1, and E-Cadherin) in the jejunum (Fig. 7, Fig. 7). Both *L. lactis* treatments effectively reversed this downregulation, with NZ-BB demonstrating superior efficacy in restoring the expression of these tight junction proteins. Cytokine profiling showed that *Salmonella* infection led to significant increases in pro-inflammatory cytokines (IL-1β, TNF-α, IL-17a, and IL-6; *P* < 0.05), while suppressing the expression of anti-inflammatory cytokines (TGF-β and IL-4; *P* < 0.05). Both probiotic treatments significantly downregulated pro-inflammatory mediators and upregulated IL-4 expression (*P* < 0.05). Notably, NZ-BB treatment induced a more pronounced upregulation of TGF-β and IL-4 compared to NZ-pNZ treatment (*P* < 0.01) (Fig. 7, Fig. 7).

**Table 5:** Flow cytometric analysis of T-cell subset changes in infected mice under NZ-BB intervention.

**DPI**	**T cells (%)**	**0.9** % **NaCl**	**0.9** % **NaCl +** **CVCC541**	**NZ-pNZ + CVCC541**	**NZ-BB +** **CVCC541**
**3**	CD^4+^	65.05	64.25	68.44	62.66
	CD^8+^	26.82	25.52	24.55	32.68
	CD^4+^/CD^8+^	2.426 ± 0.012	2.518 ± 0.013	2.788 ± 0.022	1.917 ± 0.014
**5**	CD^4+^	63.99	59.70	53.50	68.35
	CD^8+^	23.11	34.80	32.53	23.61
	CD^4+^/CD^8+^	2.769 ± 0.025**^a^**	1.715 ± 0.018	1.645 ± 0.037	2.895 ± 0.016**^a,b^**
**7**	CD^4+^	67.89	55.44	52.53	64.65
	CD^8+^	24.80	36.35	41.71	28.52
	CD^4+^/CD^8+^	2.737 ± 0.012**^a^**	1.525 ± 0.012	1.259 ± 0.007	2.267 ± 0.006**^a,b^**

Note: *P* < 0.05 indicates statistical significance; ^a^ represents 0.9 % NaCl vs 0.9 % NaCl + CVCC541, and ^b^ represents NZ-pNZ + CVCC541 vs NZ-BB + CVCC541.

### Probiotic potential of recombinant strain NZ-BB in chicks

**Fig. 8:**
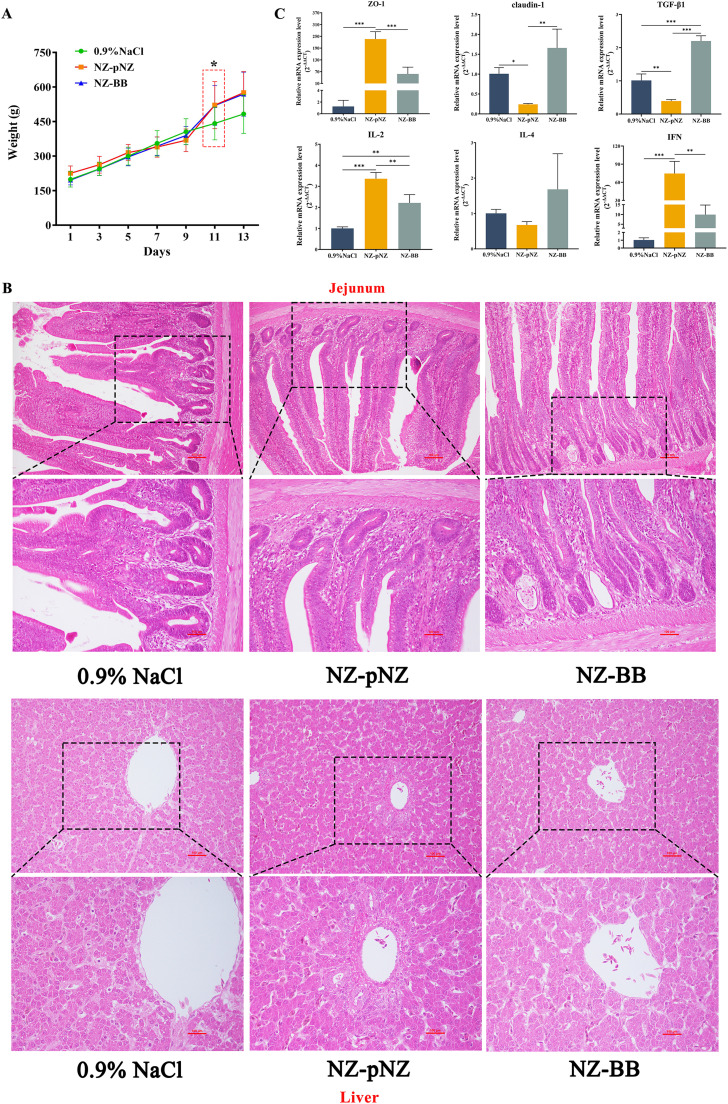
**Probiotic potential of NZ-BB in chicks.** (A) Variation of body weight. (B) Histological analysis by H&E staining. Upper panel: 200× magnification; lower panel: 400× magnification. (C) Protein expression changes assessed by RT-qPCR. Note: *P < 0.05, **P < 0.01, ***P < 0.001 indicate significant differences.

To investigate the probiotic properties and safety of recombinant strain NZ-BB in chicks, an animal trial was conducted. As shown in Fig. 8A, body weight measurements over a two-week period revealed that after 11 days of *L. lactis* administration, chicks in both the NZ-pNZ and NZ-BB groups exhibited significantly higher body weight compared to the 0.9 % NaCl group (*P* < 0.05). No statistically significant difference in body weight changes was observed between the two *L. lactis* groups (*P* > 0.05). Histopathological examination of jejunal and liver tissues via H&E staining demonstrated normal tissue architecture in both probiotic-treated groups (Fig. 8B). RT-qPCR analysis of jejunal tight junction proteins and inflammatory factors revealed distinct regulatory patterns (Fig. 8C). Both *L. lactis* strains significantly upregulated ZO-1, IL-2, and IFN expression (*P* < 0.001), with NZ-pNZ showing a more pronounced effect. Notably, NZ-pNZ treatment significantly decreased claudin-1 and TGF-β1 expression, whereas NZ-BB demonstrated the opposite effect, significantly enhancing their expression levels.

### Evaluation of the In Vivo protective effects of NZ-BB intervention against *Salmonella Pullorum* infection in chicks

**Fig. 9:**
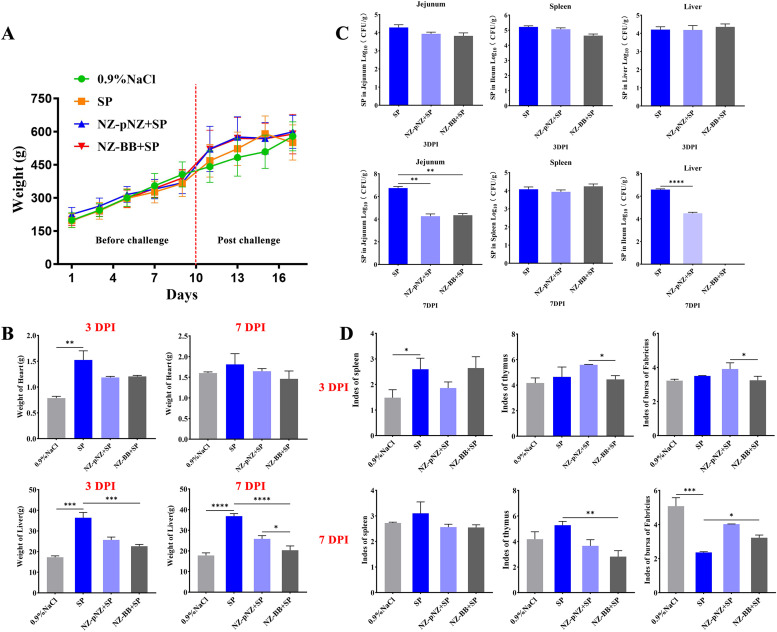
**Effects of NZ-BB intervention on body weight, physiological parameters, and immune organs in SP-infected chicks.** (A) Variation of body weight. (B) Changes in heart and liver weight. (C) SP bacterial load in jejunum, spleen, and liver. (D) Immune organ indices for spleen, thymus, and bursa of Fabricius. Note: *P < 0.05, **P < 0.01, ***P < 0.001 indicate significant differences.

**Fig. 10:**
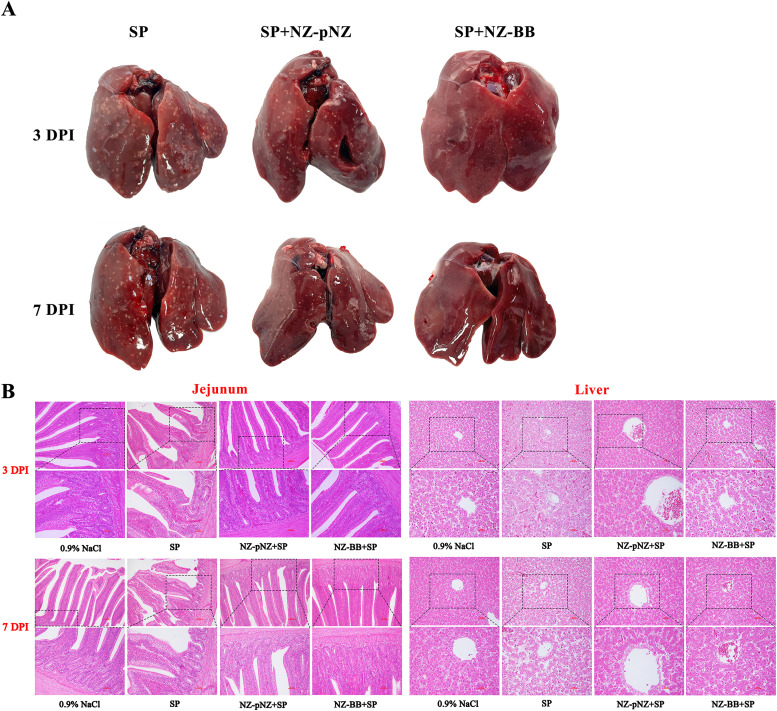
**NZ-BB intervention improves liver and jejunal histopathological changes induced by SP infection in chicks.** (A) Gross observation of liver pathological changes. (B) Histopathological analysis by H&E staining. Note: Upper panel magnification 200×; lower panel magnification 400×.

To further elucidate the protective effect of recombinant NZ-BB against SP infection in chicks, an animal trial was conducted. Body weight monitoring up to day 17 post-hatching (Fig. 9A) showed no significant differences among the four groups, indicating SP infection and probiotic treatments did not affect growth. Organ weight analysis (Fig. 9B) revealed tissue-specific responses: SP infection transiently increased heart weight at 3 DPI (*P* < 0.01) but had no effect at 7 DPI. Liver weight was significantly elevated by SP infection, which was effectively reduced by NZ-BB treatment; at 7 DPI, liver weight in the NZ-BB group was significantly lower than in the control (*P* < 0.05). Bacterial load measurements (Fig. 9C) showed similar SP colonization in all infected groups at 3 DPI. By 7 DPI, both *L. lactis* strains significantly decreased bacterial loads in jejunum and liver, with NZ-BB achieving complete clearance in the liver. Immune organ analysis (Fig. 9D) indicated a marked reduction in bursa of Fabricius index due to SP infection at 7 DPI (*P* < 0.001), partially restored by both probiotics, with no significant difference between them. Spleen and thymus indices remained unchanged. Gross pathology (Fig. 10A) at 3 DPI revealed multiple white necrotic nodules on livers in all infected groups. By 7 DPI, livers from NZ-BB-treated chicks appeared smooth and lesion-free. Histopathology (Fig. 10B) confirmed SP-induced tissue damage was significantly ameliorated by both probiotics. Jejunal villi were preserved, mucosal damage reduced, and inflammatory infiltration decreased. In the liver, hepatocyte arrangement improved, vacuolar degeneration and necrosis were diminished, and inflammation was lessened.

### Molecular mechanisms of NZ-BB intervention in protecting chicks against *Salmonella Pullorum* infection

**Fig. 11:**
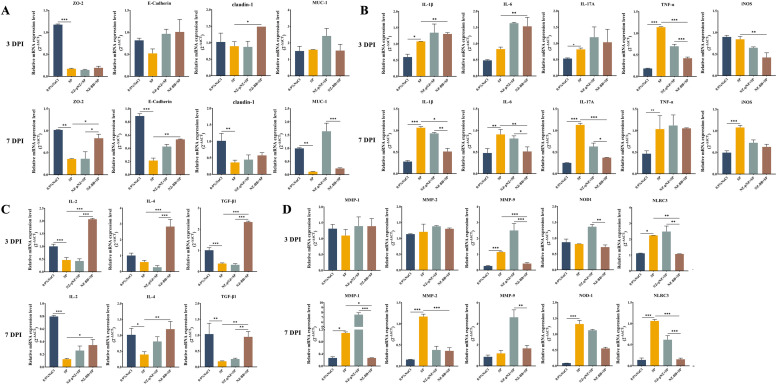
**The effect of NZ-BB on immune response and intestinal barrier function in SP-infected chicks.** Transcriptional levels of (A) barrier function-related proteins, (B) pro-inflammatory cytokines, (C) anti-inflammatory cytokines, and (D) matrix remodeling and pattern recognition receptor proteins measured by RT-qPCR. *P < 0.05, **P < 0.01, ***P < 0.001 indicate significant differences.

This study employed RT-qPCR to evaluate the effects of recombinant NZ-BB on intestinal barrier-associated proteins, pro- and anti-inflammatory cytokines, matrix-remodeling enzymes, and pattern-recognition receptors in SP-infected chicks. At 3 DPI (Fig. 11A), no significant differences were observed in the expression of tight junction proteins (ZO-2, E-Cadherin, claudin-1) or mucin MUC-1 among treatment groups. However, by 7 DPI, SP infection significantly downregulated these proteins, whereas NZ-BB administration markedly upregulated ZO-2 and E-Cadherin expression. Notably, MUC-1 expression was elevated in the SP + NZ-pNZ group compared to the infected control, warranting further investigation. Regarding pro-inflammatory cytokines (Fig. 11B), SP infection significantly increased IL-1β, IL-17A, and TNF-α levels at 3 DPI, with NZ-BB selectively suppressing TNF-α expression. By 7 DPI, both probiotic treatments significantly attenuated IL-1β, IL-6, and IL-17A expression, with NZ-BB exhibiting superior efficacy; however, TNF-α levels remained comparable between NZ-BB and infected groups. For anti-inflammatory cytokines (IL-2, IL-4, TGF-β1) (Fig. 11C), NZ-BB induced higher expression at 3 DPI relative to controls, a response meriting further exploration. At 7 DPI, infection reduced these cytokines, while both probiotics restored their expression, particularly TGF-β1, with NZ-BB demonstrating enhanced effects. Analysis of matrix remodeling proteins and pattern recognition receptors (Fig. 11D) revealed no significant differences in MMP-1 and MMP-3 expression at 3 DPI; however, SP induced a marked increase in MMP-9, which was significantly downregulated by NZ-BB (*P* < 0.001). A similar trend was observed for NLRC3 expression. At 7 DPI, infection upregulated MMP-1, MMP-2, NOD-1, and NLRC3, but only MMP-1 and NLRC3 were significantly suppressed by NZ-BB, with greater efficacy compared to NZ-pNZ.

## Discussion

Currently, bacterial expression systems dominate the production of AMPs, with *Escherichia coli* and *Bacillus subtilis* as the most commonly used prokaryotic hosts (Yi et al., 2015, Wang et al., 2013). Compared to *E. coli, B. subtilis* offers key advantages such as the absence of endotoxins, enhancing biosafety, and a highly efficient protein secretion system that facilitates correct folding of heterologous proteins (Guan et al., 2016). Likewise, *L. lactis* expression systems show great promise in AMP production due to their safety profile and probiotic properties. For instance, a recombinant *Lactobacillus casei* 393 strain carrying plasmid pPG:612-PR39 effectively expressed the PR39 and significantly reduced mortality in mice infected with enterotoxigenic *E. coli* K88+, demonstrating notable therapeutic potential (Zhang et al., 2016). In AMP engineering, the “hybridization” technique—combining two distinct AMPs to create hybrid peptides with novel properties—has attracted considerable attention (Deo et al., 2022, Deng et al., 2017). This approach offers multiple benefits: hybrid peptides are generally easier to produce at scale, often exhibit enhanced antimicrobial activity via synergistic effects, and possess improved structural stability by integrating features of both parent peptides (Sun et al., 2019, Almaaytah et al., 2018). Moreover, hybridization can reduce cytotoxicity toward eukaryotic cells. A prominent example is the fusion of Cecropin A and Forlicidin-2, which resulted in a hybrid peptide with higher production yield, strong antimicrobial efficacy, and lowered host cell toxicity, illustrating the practical value of this strategy (Yang et al., 2020b).

In this study, a fusion AMP was engineered by conjugating BMAP-18 and BSN-37 with a flexible (GGGGS)_3_ linker to enhance structural stability and ensure proper folding. The incorporation of this linker also increased the molecular weight of the target protein, thereby facilitating improved synthesis and sustained bioactivity within the expression system. The resultant fusion peptide, BMAP18-BSN37, combined the broad-spectrum antimicrobial properties of its parent peptides and exhibited enhanced potency. Furthermore, to enable extracellular secretion of the fusion protein, the signal peptide Usp45 was positioned upstream of the AMP sequence. This approach facilitated the secretion of the fusion protein from *L. lactis* host cells, thus mitigating its potential cytotoxic effects on the producer strain and enhancing both cell viability and expression stability (Hernandez-Valdes et al., 2020).

*L. lactis*, recognized as a significant probiotic, have been extensively documented to perform diverse functions in modulating inflammatory responses and augmenting host immunity. For example, *L. lactis* NK34 has been shown to markedly suppress the production of pro-inflammatory mediators, including TNF-α, IL-18, and COX-2, in LPS-stimulated macrophages RAW 264.7 (Han et al., 2015). Beyond their immunomodulatory effects, *L. lactis* contribute to the preservation of intestinal barrier integrity. They facilitate the synthesis of mucus layers that cover intestinal epithelial cells, serving as a primary defense against pathogenic invasion (Ren et al., 2018, Ohland and Macnaughton, 2010). Additionally, *L. lactis* can enhance epithelial barrier function by upregulating the expression of tight junction proteins. For instance, *L. plantarum* F3-2 significantly elevated the mRNA levels of ZO-1, occludin, and claudin-1, thereby strengthening epithelial cell-cell junctions (Bu et al., 2022). AMPs, integral components of the innate immune system, are generally expressed at low basal levels but can be rapidly upregulated in response to infection or inflammation. These peptides play a crucial role in maintaining mucosal homeostasis by suppressing pro-inflammatory cytokines and enhancing the expression of tight junction proteins (Ma et al., 2020). For instance, LL-37 safeguarded colonic epithelial integrity by modulating TLR4 signaling and curtailing excessive IL-1β production (Marin et al., 2019). Among the characterized AMPs, BMAP-18 and BSN-37 exhibit both significant antimicrobial activity and notable immunomodulatory properties. BMAP-18 had been shown to inhibit NO production and TNF-α release in LPS-stimulated RAW264.7 (Lee et al., 2011), whereas BSN-37 enhances the secretion of Th1-type cytokines, such as IL-2 and IFN-γ, as well as Th2-type cytokines, including IL-10 and IL-4 (Li et al., 2023).

In this study, recombinant NZ-BB strain was designed to express a fusion AMP comprising BMAP-18 and BSN-37, with the objective of achieving synergistic effects surpassing the additive effects of the individual peptides. The probiotic characteristics and biosafety of NZ-BB were assessed using both murine and avian models. In murine models, NZ-BB significantly upregulated the expression of occludin and claudin-1, thereby enhancing the integrity of the intestinal barrier. Additionally, it suppressed the expression of IL-1β while promoting the expression of anti-inflammatory cytokines such as TGF-β, IL-4, and IL-6. In avian models, NZ-BB similarly increased the expression of ZO-1, claudin-1, and TGF-β1, further corroborating its role in fortifying mucosal barriers and modulating immune responses.

*Salmonella* is a common foodborne pathogen, capable of breaching the intestinal epithelial barrier and triggering inflammatory responses, which can result in diarrhea, or increased mortality in young animals, thereby posing significant risks to animal health and food safety (Crump et al., 2015). AMPs have garnered significant interest due to their extensive antibacterial activity. Recently, certain strains of *L. lactis* have been identified to possess inherent antimicrobial properties. For example, *L. lactis* JNU 534 has been documented to mitigate the adverse effects of *Salmonella* infection on broiler performance, enhancing growth metrics without adversely affecting meat quality or animal health (Purnamasari et al., 2025). In this study, a systematic evaluation was conducted to investigate the antimicrobial efficacy and underlying mechanisms of the recombinant strain NZ-BB against *Salmonella* infection in both murine and avian models. In mice, NZ-BB significantly upregulated the expression of intestinal tight junction proteins, including ZO-1, occludin, claudin-1, and E-cadherin in the jejunum, thereby contributing to the preservation of intestinal barrier integrity. Furthermore, NZ-BB modulated host immune responses by significantly downregulating pro-inflammatory cytokines, including IL-1β, TNF-α, and IL-17A, while upregulating anti-inflammatory mediators such as TGF-β and IL-4. In the chick model, molecular analyses demonstrated that NZ-BB intervention substantially enhanced the expression of intestinal barrier proteins ZO-2 and E-cadherin. Additionally, it modulated the levels of both pro-inflammatory (IL-1β, IL-6, IL-17A) and anti-inflammatory (TGF-β1) cytokines. Importantly, NZ-BB also significantly suppressed the expression of inflammation-associated markers, such as MMP-9 and NLRC3, thereby reinforcing its protective role against *Salmonella*-induced intestinal inflammation.

At the experimental design stage, this study focused on the core research priority of evaluating the immunomodulatory and anti-infection functions of the recombinant strain NZ-BB in mice and chicks, with an emphasis on its intestinal barrier enhancement and inflammatory factor regulatory effects. Therefore, the determination of production performance parameters, feed consumption and efficiency parameters, as well as health and welfare-related indicators was not incorporated. The aforementioned indicators represent a key dimension for comprehensively verifying the application value of probiotics in animal husbandry production. In future research, we will optimize the experimental protocol, systematically supplement and determine the relevant parameters, so as to further improve the application potential evaluation system of this strain.

In conclusion, NZ-BB engineered to express the fusion antimicrobial peptide BMAP18-BSN37 was successfully developed and characterized. This strain demonstrated stable expression of the target protein and maintained high plasmid stability. In both murine and broiler chicken models, NZ-BB significantly enhanced growth performance, improved immune organ function, fortified intestinal barrier integrity, and modulated the expression of inflammatory cytokines. In *Salmonella* infection models, NZ-BB effectively mitigated pathological damage, reduced bacterial loads in tissues, and achieved pathogen clearance in specific organs by days 5 or 7 post-infection. Overall, NZ-BB exhibits promising probiotic properties, as well as immunomodulatory and anti-infective activities, underscoring its potential as an innovative functional probiotic candidate.

## Author Contributions

Lei Wang and Ruibiao Wang designed this study. Ruibiao Wang, Yukai Lin, Yu Xia, Suxian Liu, Doudou Feng, Siyang Li, Tengyue Zhou, Huarun Sun, Jiyuan Shen, Bo Wen, Minghui Li, Chengshui Liao, Baoliang Qin, Jianhe Hu, Ke Ding performed the experiments. Ruibiao Wang drafted and revised the manuscript. All authors read and approved the final manuscript.

## Funding

This work was supported by the Young Scientists Fund of the Natural Science Foundation of Henan (252300421655); Key Science and Technology Research of Henan (242102110031); National Natural Science Foundation of China (32172862, 32473037); National Key R&D Program of China (2021YFD1301200); Science and Technology Innovative Research Team in Higher Educational Institutions of Henan Province (24IRTSTHN035); the Joint Fund of Science and Technology Research and Development Plan in Henan (225200810044); Key Research and Development Project of Henan Province (241111110100).

## Availability of data and materials

The datasets used or analyzed during the current study are available from the corresponding author on reasonable request.

## Ethics approval and consent to participate

The experiment was approved by the Laboratory Animal Welfare and Ethics Committee of Henan Institute of Science and Technology and was conducted in accordance with ethical guidelines and the approved protocol (Ethics Approval No. LLSC2024052).

## CRediT authorship contribution statement

**Ruibiao Wang:** Writing – review & editing, Writing – original draft, Methodology, Investigation, Funding acquisition, Data curation. **Yukai Lin:** Investigation. **Yu Xia:** Investigation. **Suxian Liu:** Investigation. **Doudou Feng:** Investigation. **Siyang Li:** Investigation. **Tengyue Zhou:** Investigation. **Huarun Sun:** Investigation. **Jiyuan Shen:** Investigation. **Bo Wen:** Investigation. **Minghui Li:** Investigation. **Chengshui Liao:** Investigation. **Baoliang Qin:** Investigation. **Jianhe Hu:** Writing – review & editing. **Yuanfang Ma:** Writing – review & editing. **Ke Ding:** Writing – review & editing. **Lei Wang:** Writing – review & editing, Funding acquisition.

## Disclosures

The authors declare that they do not have any commercial or associative interest that represents a conflict of interest in connection with the work submitted.
